# Implantation with SHED sheet induced with homogenate protein of spinal cord promotes functional recovery from spinal cord injury in rats

**DOI:** 10.3389/fbioe.2023.1119639

**Published:** 2023-03-14

**Authors:** Sisi Mi, Xue Wang, Jiaxin Gao, Yu Liu, Zhongquan Qi

**Affiliations:** Medical College, Guangxi University, Nanning, China

**Keywords:** spinal cord injury, cell sheet technology, spinal cord homogenate, neurotrophins, neuroplasticity, regeneration

## Abstract

**Introduction:** After spinal cord injury (SCI) occurs, the lesion is in a growth inhibitory microenvironment that severely hinders neural regeneration. In this microenvironment, inhibitory factors are predominant and factors that promote nerve regeneration are few. Improving neurotrophic factors in the microenvironment is the key to treating SCI.

**Methods:** Based on cell sheet technology, we designed a bioactive material with a spinal cord‐like structure –SHED sheet induced with homogenate protein of spinal cord (hp–SHED sheet). Hp–SHED sheet was implanted into the spinal cord lesion for treating SCI rats with SHED suspensions as a control to investigate the effects on nerve regeneration.

**Results:** Hp–SHED sheet revealed a highly porous three–dimensional inner structure, which facilitates nerve cell attachment and migration. Hp-SHED sheet *in vivo* restored sensory and motor functions in SCI rats by promoting nerve regeneration, axonal remyelination, and inhibiting glial scarring.

**Discussion:** Hp–SHED sheet maximally mimics the microenvironment of the natural spinal cord and facilitate cell survival and differentiation. Hp–SHED sheet could release more neurotrophins and the sustained action of neurotrophins improves the pathological microenvironment, which effectively promotes nerve regeneration, axonal extension, and inhibits glial scarring, thereby promoting the *in situ* centralis neuroplasticity. Hp–SHED sheet therapy is a promising strategy for effective treatment of SCI based on neurotrophins delivery.

## 1 Introduction

Spinal cord injury (SCI) is a serious and highly disabling disease of the central nervous system. More than 20 million patients worldwide currently suffer from SCI, with an annual increase of approximately 700,000 individuals (Global et al., 2018). SCI is divided into primary and secondary injuries. Primary injury directly causes neuron death and axon disruption. Secondary injury involves a series of biological cascades caused by primary injury, such as inflammatory responses, apoptosis, gray matter breakdown, white matter demyelination, and glial scar formation (Tran et al., 2018; Yu and Fehlings, 2011). Essentially, secondary injury is a complex process of multicellular and multimolecular interactions (Rubiano et al., 2015). The lesions are infiltrated by a large number of dead cells as well as inflammatory cells, while microglia activation transforms into macrophages to phagocyte debris and degenerate myelin sheaths (Lin et al., 2021) and astrocytes proliferate to form glial scars. The glial scar is a physical barrier to nerve regeneration and axonal growth (Anderson et al., 2016; Dias et al., 2018). The factors above allow the establishment of a local growth inhibitory microenvironment within the lesion, which severely hinders nerve regeneration after SCI. Moreover, the intrinsic repair capacity of the central nervous system is highly limited. Current clinical therapies are not yet able to achieve effective functional recovery and it is difficult to achieve effective neurological recovery by targeting a single factor (Thuret et al., 2006). Thus, comprehensive regulation of the lesion microenvironment is key for SCI treatment (Li et al., 2019; Wang et al., 2021).

Stem cells from human exfoliated deciduous teeth (SHED) are mesenchymal stem cells (MSCs) derived from neural crest, which is homologous to central nervous system tissue (Miura et al., 2003). Compared with MSCs from other sources, SHED display stronger neurotropic characteristics, including the ability to migrate to sites of neural injury and differentiate into neurons and oligodendrocytes (Hochuli et al., 2021; Huang et al., 2009). Accordingly, SHED have been used to treat a variety of neurological diseases (Fujii et al., 2015; Huang et al., 2022; Pereira et al., 2019). Currently, SHED are used for treatment of SCI with some efficacy (Nicola et al., 2017; Sakai et al., 2012). The therapeutic neurotrophic effects of SHED have been attributed to a paracrine role *via* the paracrine factors including cytokines, growth factors, immune-modulatory proteins, and exosomes (Chen et al., 2020; Sugimura-Wakayama et al., 2015).

The type of cells that MSCs differentiate into is closely related to the microenvironment in which they live (Tang et al., 2020). The microenvironment in which the cells of different tissues are located differs, most notably in various factors. This is one of the main reasons for the differentiation of stem cells to other tissue cells (Hwang et al., 2009). The component of spinal cord homogenate is complex and includes some chemical substances or cytokines. Previous studies showed that spinal cord homogenate could be used as an effective inducer to induce BMSCs to differentiate into neuronal cells and secrete brain-derived neurotrophic factor (Liu et al., 2011; WU et al., 2009). Studies previously found that inoculation of DCs pulsed with homogenate proteins of the spinal cord (hpDCs) promotes functional recovery from SCI in mice. Application of hpDCs can improve hindlimb motor function after SCI in rats by increasing the expression of neurotrophic factors and cytokines at the injury site (Liu et al., 2009; Wang et al., 2013).

Cell sheet technology (CST) refers to the inoculation of high-density cells *in vitro*, culturing them and growing them in layers to form a dense membrane-like structure rich in cells and extracellular matrix (ECM) (Yang et al., 2005). CST completely retains the rich signal transmission between cells and their ECM, and can mimic the *in vivo* microenvironment to maximize the biological properties of cells (Elloumi-Hannachi et al., 2010; Lin et al., 2013). Compared to cell suspensions, CST facilitates cell survival, migration, proliferation, and differentiation (Lin et al., 2013; Takeuchi et al., 2016). Nowadays, CST has become a popular research topic in the field of tissue engineering, and been successfully applied to the repair of periodontal (Iwata et al., 2018), cornea (Nishida et al., 2004), bone (Liu et al., 2013), cartilage (Sato et al., 2014), esophagus (Ohki et al., 2012), heart (Shimizu et al., 2003), and other tissues. In addition, CST has been shown to promote nerve regeneration and functional recovery after SCI (Fan et al., 2020; Yamazaki et al., 2021).

Based on the above techniques, a scheme is proposed. SHED sheet is cultured with homogenate proteins of the spinal cord (hp-SHED sheet). In the microenvironment rich in spinal cord homogenate, SHED differentiate into neural cells. Hp-SHED sheet can release more cytokines and neurotrophins with neuroprotective functions. In this project, hp-SHED sheet was implant into the spinal cord lesion for treating SCI rats with SHED suspensions as a control to investigate the effects on neural regeneration and repair.

## 2 Results

### 2.1 Cell identification

**FIGURE 1:**
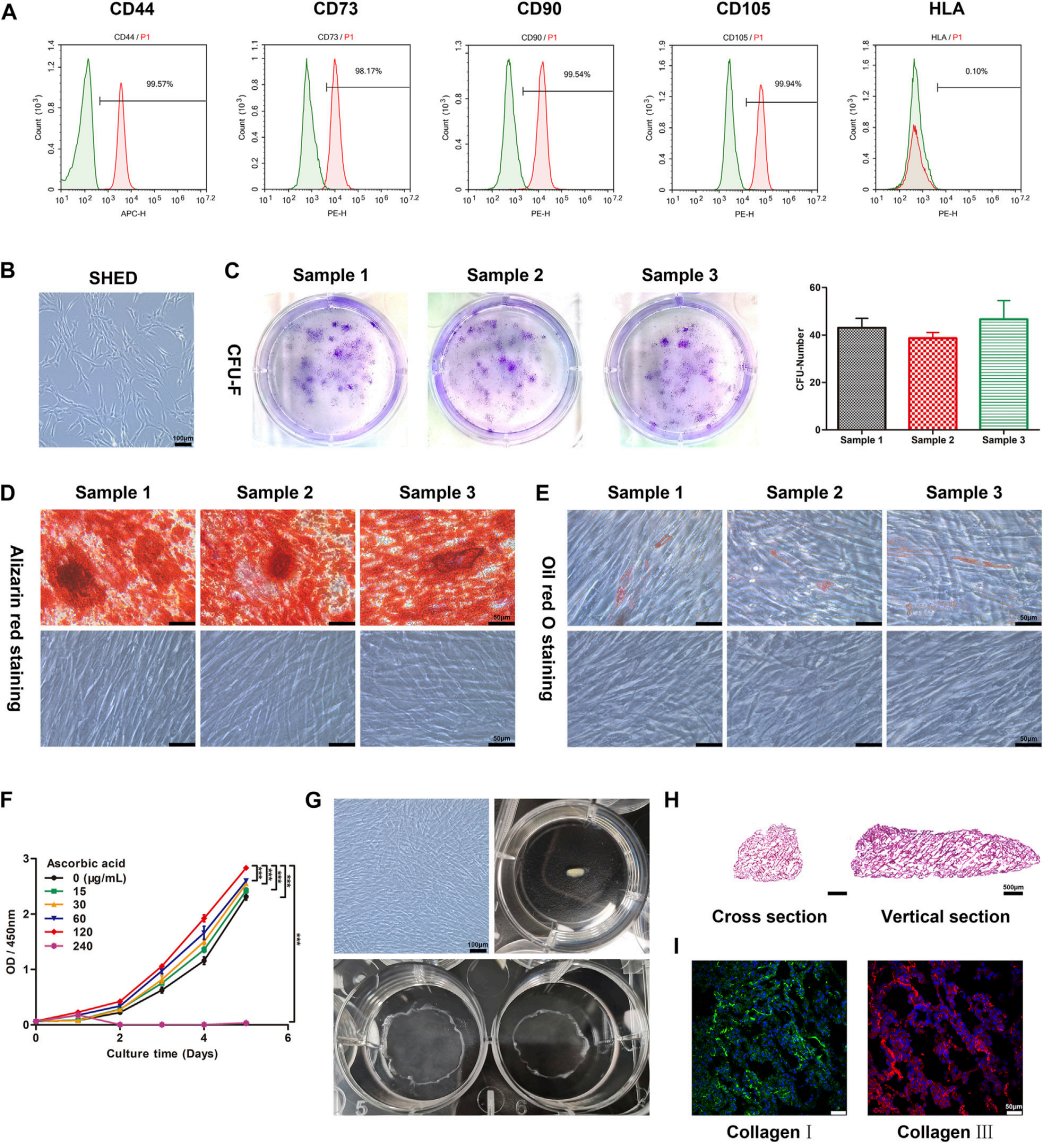
The construction and characteristics of hp-SHED sheet, which possesses a geometric structure similar to that of spinal cord tissue. **(A)** The surface markers of SHED, including CD44, CD73, CD90, CD105 and HLA were analyzed by flow cytometry. **(B)** Map of the morphology of the SHED. **(C)** SHED formed single CFU clusters in culture, the quantitative result was indicated in right panel. Data shown as mean ± SD. **(D)** Osteogenic differentiation of SHED. Alizarin red staining was performed at day 21 after osteogenic induction. **(E)** Adipogenic differentiation of SHED. Oil Red O staining was performed at day 28 after adipogenic induction. **(F)** The proliferation curve of SHED with different concentrations of ascorbic acid (AA) was analyzed by CCK8 assay. (mean ± SD, ***p < 0.001). **(G)** Construction of hp-SHED sheet. **(H)** Representative images of hp-SHED sheet sections. **(I)** Collagens in hp-SHED sheet were characterized by CLSM.

SHED were obtained from donors. The morphology of cells was irregular, mostly spindle or polygonal shapes. The cell bodies were small, the cytoplasm was abundant and transparent, and there were almost no protrusions (Figure 1B). The identity of SHED was confirmed by evaluating expression of specific marker proteins by flow cytometry. Percentages of cells expressing positive markers CD44, CD73, CD90, and CD105 were 99.57%, 97.27%, 99.54%, and 99.91%, respectively. Furthermore, the percentage of cells expressing the negative marker human leukocyte antigen was 0.10%, indicating successful isolation of SHED (Figure 1A). In a colony-formation assay, SHED proliferated after 10 days and cell clone formation was visible under light microscopy. After crystalline violet staining, multiple cell colonies were visible (Figure 1C). Following induction of osteogenic differentiation of SHED for 21 days, calcium nodule deposition was visible and Alizarin Red staining was positive (Figure 1D). Following induction of lipogenic differentiation of SHED for 28 days, lipid droplets were observed in the cytoplasm, which stained positive for Oil Red O (Figure 1E). In summary, the extracted cells were SHED with the potential for unlimited proliferation, osteogenic differentiation, and lipogenic differentiation.

### 2.2 Hp-SHED sheet preparation and characterization

To optimize the formation of SHED sheet, we assayed the effect of varying AA concentrations on the proliferation of SHED. The results show that 240 μg/mL AA obviously inhibited proliferation, and while 120 μg/mL AA promoted cell proliferation and facilitated cell sheet formation. Thus, we chose 120 μg/mL AA for cell sheet construction (*p* < 0.001, Figure 1F). After SHED were cultured with complete medium containing AA for 1 week, SHED sheet was incubated with homogenate protein of spinal cord for 1 week to differentiate into neural cells. Finally, hp-SHED sheet peeled off the culture dish and self-assembled into a spinal cord-like shape, forming the bioactive filling material (Figure 1G).

First, hp-SHED sheet can serve as a biological scaffold, providing a structural basis for cell growth, differentiation, migration, and axonal extension. Cross and vertical sections of hp-SHED sheet revealed a highly porous three-dimensional inner structure, which facilitates nerve cell attachment, migration, and nerve axon regeneration and extension (Figure 1H). In addition, our results show that both collagens Ⅰ and Ⅲ were positively expressed in hp-SHED sheet. Cells in the hp-SHED sheet were tightly arranged, had a “honeycomb” structure, and regular ECM distribution (Figure 1I). High expression of collagen provides stability to the ECM microenvironment, enhancing intercellular interactions within hp-SHED sheet. Stable ECM is essential for sustained and stable cytokines and neurotrophins production in hp-SHED sheet.

### 2.3 Hp-SHED sheet effectively restored sensory and motor functions in SCI rats

**FIGURE 2:**
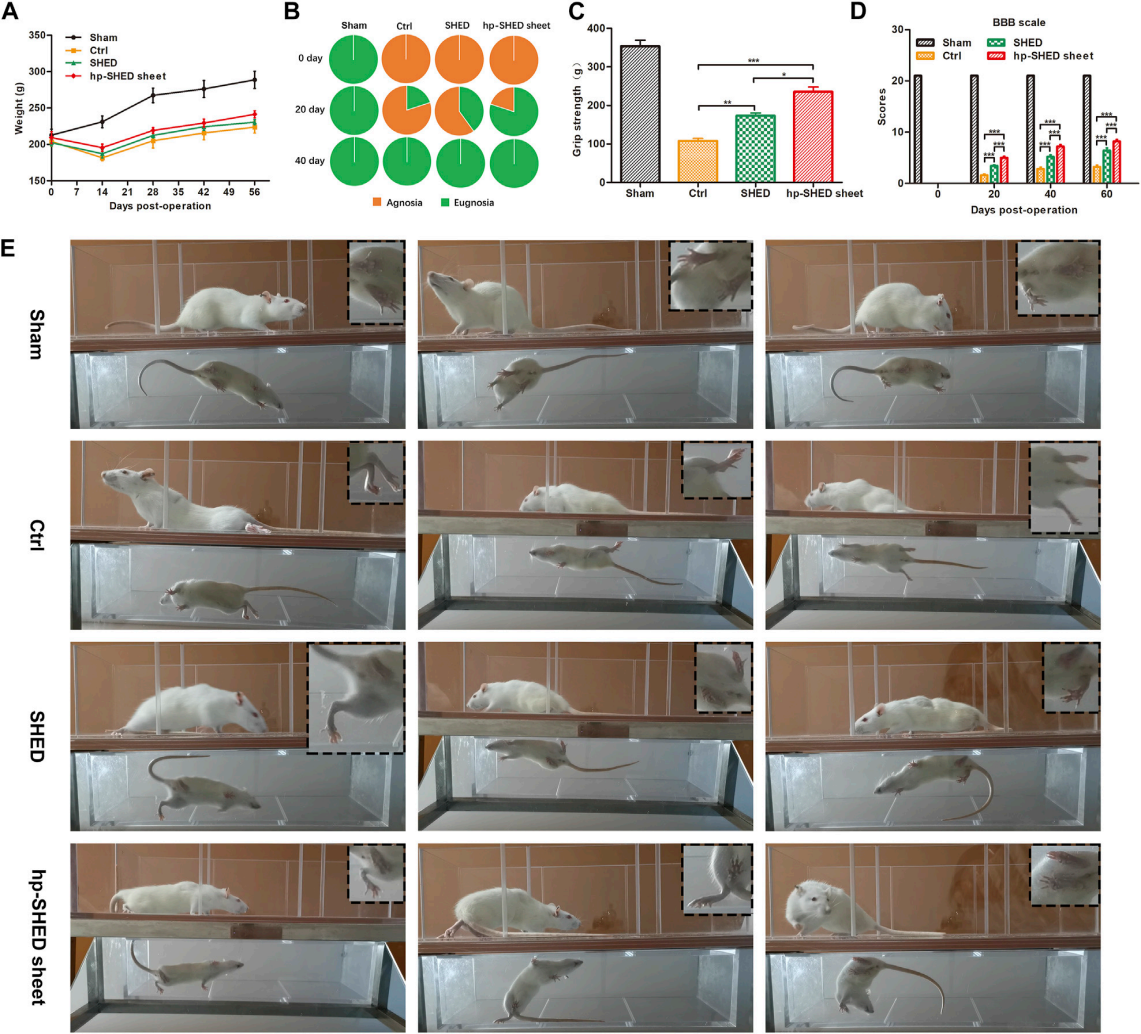
Hp-SHED sheet was more active than SHED to recover the sensory and motor in complete SCI rats. **(A)** The weight change in SCI rat model with indicated treatments. **(B)** The percentage of sensory restorative rats at days 0, 20, and 40 post-treatment. **(C)** Grip strength of each group at 60 days post-treatment. **(D)** BBB scores of sham operated rats (Sham) and complete SCI rats upon PBS (Ctrl), SHED, or hp-SHED sheet treatment. **(E)** Representative crawling photos of rats in the indicated group at day 60 post-treatment. (mean ± SD, *p < 0.05, **p < 0.01, ***p < 0.001).

To systematically and comprehensively explore the biological functions of hp-SHED sheet, 20 rats were divided into four groups: Sham, Control (Ctrl), SHED, and hp-SHED sheet (*n* = 5 per group). The spinal cord at T9–T10 was cut in rats to establish a complete SCI model. After modeling, the lesion cavity was filled with SHED or hp-SHED sheet in experimental groups, and PBS in the Ctrl group (Supplementary Figure S1). During the 60-day recovery period, all rats were subjected to body weight measurements, Von Frey testing, BBB scoring and grip strength test every 20 days. Before surgery, rats in each group were weighed. After surgery, rats lost weight because of the effects of spinal cord trauma. However, several days later, the weight of rats started to recover and gradually increased. Weight recovery was faster and more evident in the hp-SHED sheet group, followed by the SHED group, and poorest in the Ctrl group. However, differences between groups were not statistically significant. The rats in the hp-SHED sheet group recovered better and had better appetite, but with an increase in locomotion. The rats in the Ctrl group recovered less and had a poor appetite, but with less locomotion due to poor recovery of the hind limbs. The SHED group was in between. Eating and energy expenditure were in balance, so there was no significant difference in body weight (Figure 2A).

Von Frey filaments were used to detect the sensory recovery of rat hindlimbs. On the second day after surgery, all three groups of SCI rats had complete sensory deficits and negative hindlimb sensory ratings; whereas, rats in the Sham group had sensitive hindlimb sensation, with all rats responding positively. Twenty days after surgery, 20% of Ctrl rats recovered sensation, 40% of SHED rats recovered sensation, and 80% hp-SHED sheet rats recovered sensation (Figure 2B). Thus, the hp-SHED sheet group had a higher proportion of rats with sensory recovery compared with the SHED group, was both were significantly higher than Ctrl group. Rats in the hp-SHED sheet group recovered earlier and more completely.

The recovery of sensation was always accompanied by the recovery of motor ability. At 60 days, average grip strengths were 107.80 g ± 14.81 g in the Ctrl group, 173.00 g ± 16.70 g in the SHED group, and 235.40 g ± 27.93 g in the hp-SHED sheet group (*p* < 0.05, Figure 2C). At 60 days, average BBB locomotor scores were 3.20 ± 0.84 in the Ctrl group, 6.40 ± 1.14 in the SHED group, and 8.20 ± 0.84 in the hp-SHED sheet group (*p* < 0.001, Figure 2D). After 60 days of treatment, Ctrl rats were still unable to crawl on their hind legs and had only slight movement of the hindlimb joints. While SHED rats showed extensive movement of the three joints in the hindlimbs. Hp-SHED sheet rats could land on the plantar placement of the paw with no weight support (Figure 2E; Supplementary Video S1). Compared with the Ctrl group, the joint movement of both hp-SHED sheet and SHED groups showed significant improvement, although the treatment effect of hp-SHED sheet was more significant. In conclusion, hp-SHED sheet produced more significant neurorestorative effects in terms of both sensory and joint movement recovery.

### 2.4 Hp-SHED sheet *in vivo* restored sensory and motor functions in SCI rats by promoting nerve fiber regeneration and axonal remyelination, and inhibiting glial scarring

**FIGURE 3:**
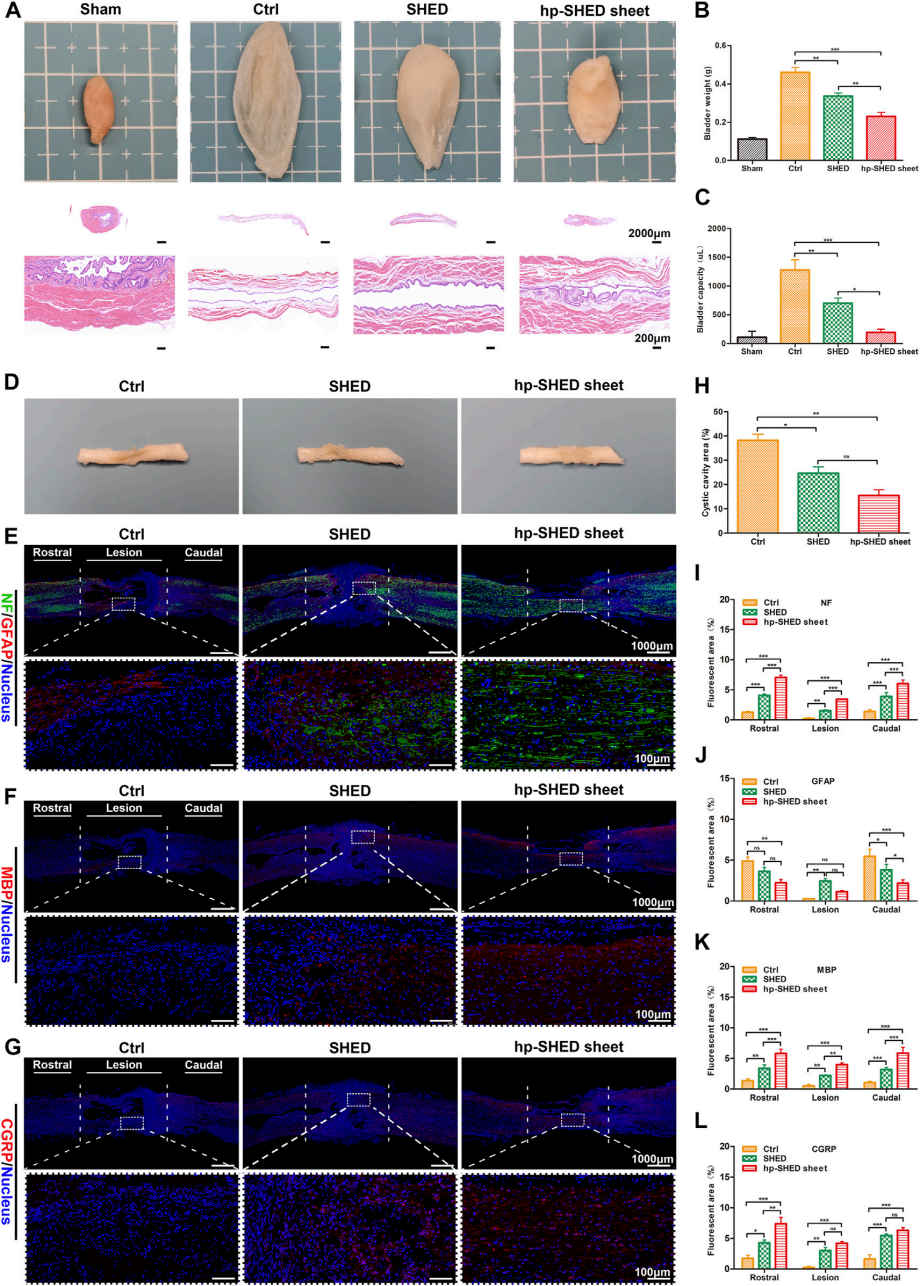
Histological mechanisms underlying sensory and motor recovery. **(A)** Representative images and HE staining of bladders from rats in the indicated group. **(B–C)** Quantification and comparison of bladder weight and capacity in the indicated groups. **(D–G)** Spinal cord representative images and IF staining of NF (green) and GFAP (red), MBP (red), CGRP (red), in Ctrl, SHED and hp-SHED sheet. Images below showed the magnified views of the lesion area that are boxed in the above images. **(H–L)** Quantification and comparison of NF, GFAP, MBP, CGRP expression and cystic cavity area. (mean ± SD, *p < 0.05, **p < 0.01, ***p < 0.001, ns = no significance).

We analyzed the recovery of spontaneous urination in rats from histological characteristics of the bladder. Compared with the Sham group, the Ctrl group had overly enlarged bladders due to urinary retention and loss of medial bladder folds, along with abnormally large bladder volumes and increased bladder weights. In contrast, following treatment with SHED or hp-SHED sheet, there were significant improvements in various aspects of bladder size, fold texture, bladder volume, and bladder weight. Notably, for all these observations, hp-SHED sheet was more effective (*p* < 0.05, Figures 3A–C). The analysis of bladder mucosal folds, weight, and capacity revealed that both hp-SHED sheet and SHED had therapeutic effects compared with the Ctrl group, although bladders in the hp-SHED sheet group were more like the Sham group. These results demonstrate that rats in the hp-SHED sheet group recovered spontaneous urination function earlier.

After spinal cord sampling at 60 days postoperatively, it was found that the recovery at the spinal cord lesion differed significantly between the groups (Figure 3D). To compare groups, pathological analysis of the spinal cord was performed. Specifically, the spinal cord lesion, as well as rostral and caudal stumps, were stained for markers of neurons (NF), astrocytes (GFAP), myelin (MBP), and regenerating peripheral nerves (CGRP) to detect neuronal regeneration, glial scar production, and neuronal axonal remyelination. The results revealed an absence of NF staining at the site of SCI in Ctrl rats, and there were obvious voids at the damage site. In SHED rats, there was a small amount of positive NF staining at the damage site. In hp-SHED sheet rats, a large amount of NF positive staining was clearly observable at the injury site, and there was no obvious cavity at the damage site (Figure 3E). Quantification of NF-positive areas at the lesion site shows that the hp-SHED sheet group had the largest area of positive neuron staining (3.43% ± 0.04%) compared to the Ctrl (0.21% ± 0.17%) and SHED groups (1.52% ± 0.16%), indicating that more neurons regenerated, matured, and formed nerve fibers in the SCI lesion this group (*p* < 0.01, Figure 3I).

High numbers of astrocytes promote glial scar deposition, which in turn prevents nerve fiber growth into and extension of nerve axons. To detect astrocyte formation, GFAP staining was performed. In Ctrl rats, the least GFAP staining was observed at the SCI lesion site because of the massive cell death (0.26% ± 0.08%) (Figure 3J). In SHED rats, positive GFAP staining was increased at the damage site as treatment inhibits cell death. In contrast, only weak GFAP-positive staining was observed in hp-SHED sheet rats because the treatment allowed more cells to survive and to differentiate towards non-astrocyte (Figure 3E). Quantification of GFAP-positive areas at the lesion site shows that hp-SHED sheet rats had less GFAP staining (1.15% ± 0.12%) than SHED rats (2.46% ± 0.31%) (Figure 3J). As shown by percentages of GFAP-positive cells, at the rostral and caudal site, astrocyte formation and glial scar deposition were reduced in SHED and hp-SHED sheet rats compared with Ctrl rats, with hp-SHED sheet having the most evident inhibitory effect. At the lesion site, besides the massive cell death in Ctrl rats, hp-SHED sheet rats effectively inhibited astrocyte production compared to SHED rats. Thus, hp-SHED sheet reduced glial scar deposition to mitigate its inhibitory effect on nerve regeneration by decreasing astrocyte production.

During the early stages of SCI, axonal demyelination occurs. Regeneration of myelin can be determined by assessing MBP expression. We did not observe MBP staining in spinal cords of Ctrl rats, while SHED rats displayed small amounts of MBP-positive staining. In hp-SHED sheet rats, large amounts of MBP-positive staining could be observed (Figure 3F). Quantification of MBP-positive areas at the lesion site shows that myelin-positive areas were significantly larger in SHED rats (2.22% ± 0.09%) than Ctrl rats (0.51% ± 0.25%), and significantly higher in hp-SHED sheet rats (3.99% ± 0.28%) than SHED rats, with hp-SHED sheet rats exhibiting the densest myelin structures (*p* < 0.01, Figure 3K).

CGRP is a marker of nerve regeneration, especially early nerve regeneration, that is often used as a rating index for spinal cord repair and regeneration. Our results show that Ctrl rats had almost no CGRP-positive cells, while CGRP-positive staining was increased in the SHED group and highest in the hp-SHED sheet group (Figure 3G). When areas of CGRP-positive staining in each group were quantified, the hp-SHED sheet group had the highest CGRP expression (4.18% ± 0.49%) compared to the Ctrl (0.25% ± 0.15%) and SHED groups (2.98% ± 0.92%) at the lesion site (Figure 3L). Increased numbers of CGRP-positive neurons in hp-SHED sheet rats suggests that hp-SHED sheet can promote neural regeneration earlier and better.

NeuN is a neuron-specific nuclear protein. NeuN staining was performed on the spinal cord, and it was found that there were a large number of neurons at the lesion in the hp-SHED sheet group. In SHED group, there were a few neurons in the lesion. The Ctrl group saw almost no neurons (Supplementary Figure S2A). When areas of NeuN-positive staining in each group were quantified, the hp-SHED sheet group had the highest NeuN expression (2.96% ± 0.69%) compared to the Ctrl (1.01% ± 0.22%) and SHED groups (1.79% ± 0.67%) at the lesion site (Supplementary Figure S2B). IBA1, a 143 amino acid cytoplasmic, inflammation response scaffold protein, is the microglia-specific marker. We stained the spinal cord for IBA1 and found a small distribution of microglia at the lesion in the hp-SHED sheet group. There were more microglia at the lesion in the SHED group. In the Ctrl group, microglial cell expression varied greatly, with some rats having significant microglia expression at the spinal cord lesions, while others had almost no positive expression due to massive cell death (Supplementary Figure S2A). When the Iba1-positive staining area was quantified for each group, the hp-SHED sheet group had the lowest Iba1 expression at the lesion site (0.68% ± 0.28%) compared to the Ctrl group (1.42% ± 1.14%) and the SHED group (1.89% ± 0.29%) (Supplementary Figure S2C).

Persistent inflammatory cell infiltration after SCI leads to further cell death and destruction of cellular and extracellular structures, resulting in the formation of cystic cavities at the lesion. After 60 days of treatment in SCI rats, spinal cord lesions displayed different degrees of cavities among the three groups. Quantification of these cavity areas reveals that hp-SHED sheet rats had the smallest average cavity area (15.49% ± 4.09%), followed by SHED rats (24.64% ± 4.58%) and Ctrl rats (38.23% ± 4.37%) which had the largest cavity area (Figure 3H). Following hp-SHED sheet treatment, more neurons, Schwann cells, and oligodendrocytes regenerated, and regenerated nerve fibers and nerve axons could extend to the lesion area, reducing the cystic area of the spinal cord lesion and promoting reconstruction of spinal cord neural circuits.

### 2.5 Hp-SHED sheet maintained a favorable safety profile

**FIGURE 4:**
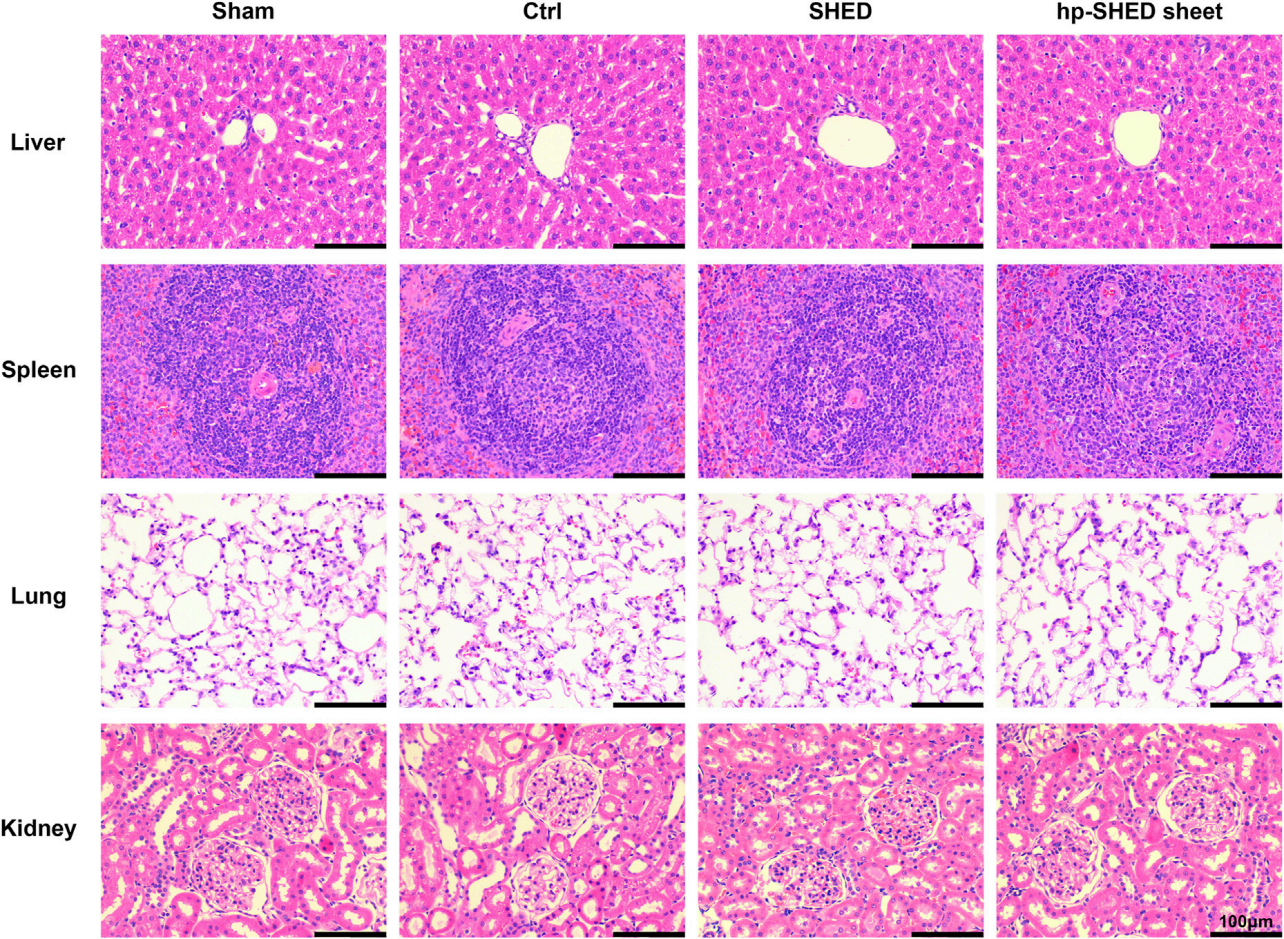
The HE staining of liver, spleen, lung and kidney in each group of rats on the 60th day postoperatively.

To further explore the potential of this bioactive filling material for clinical applications, we examined potential histological changes of several major organs of rats after transplantation. As expected, histopathological sections of the liver, spleen, lung, and kidney showed no abnormalities by HE staining, indicating that hp-SHED sheet and SHED are non-immunotoxic and preliminarily safe as human filler materials (Figure 4).

## 3 Discussion

After SCI occurs, the lesion is in a growth inhibitory microenvironment that severely hinders neural regeneration. In this microenvironment, inhibitory factors are predominant and factors that promote nerve regeneration are few (Rubiano et al., 2015). Some evidence suggests that neurotrophic cytokines and neurotrophins play a dominant role in the protection of the nervous system (Moalem et al., 2000). Improving neurotrophic factors in the microenvironment is the key to treating SCI. Therefore, an effective delivery strategy to achieve high retention and sustained release of neurotrophins into spinal cord lesion is urgently needed.

MSC transplantation replenishes the large number of cells that die after the occurrence of SCI and has a therapeutic effect. However, numerous studies have shown that cell survival is low after MSC transplantation, and it is the paracrine secreted neurotrophic factors that play the main therapeutic role (Chen et al., 2020; Sugimura-Wakayama et al., 2015). CST overcomes the problem of low survival rate of single-cell suspension. With CST, the cells remain active and are able to continuously secrete neurotrophic factors by paracrine (Yan et al., 2022). Indeed, the cell sheet can be regarded as a structural platform for natural, stable, and sustained release of neurotrophins. In addition, compared to cell suspensions, CST facilitates cell migration, proliferation, and differentiation (Lin et al., 2013; Takeuchi et al., 2016). The component of spinal cord homogenate proteins is complex and includes some chemical substances or cytokines. Previous studies showed that spinal cord homogenate proteins could be used as an effective inducer to induce BMSCs to differentiate into neuronal cells and secrete brain-derived neurotrophic factor (Liu et al., 2011; WU et al., 2009). In this study, SHED sheet was induced with homogenate proteins of the spinal cord to induce more cytokines and neurotrophins production for treating SCI mice with SHED suspensions as a control.

Hp-SHED sheet can serve as a biological scaffold, providing a structural basis for cell growth, differentiation, migration, and axonal extension. Ascorbic acid (AA) has been shown to promote intercellular adhesion *via* collagen to stimulate SHED self-assembly into flexible cell sheet structures (Yan et al., 2022). Collagen has good biocompatibility and degradability, and its collagen-binding domain and ordered structure provide a suitable basis for accommodating neurotrophic factors within a certain area, thus guiding neuronal axons to grow in a certain direction in an orderly manner (Li and Dai, 2018). In summary, hp-SHED sheet maximally mimics the microenvironment of spinal cord development.

Hp-SHED sheet not only serves as a biological scaffold to provide a structural basis for neural cell regeneration and axonal extension. It also secretes various neurotrophic factors related to nerve growth through the paracrine pathway. In the lesion microenvironment of SCI, neural stem cells were more inclined to differentiate into astrocytes and form scar tissue, which inhibits nerve cell regeneration and the reconstruction of neural circuits (Cao et al., 2001; Wang et al., 2008). SHED may act as neuroprotective agents after transplantation, possibly through paracrine signaling, to reduce glial scar formation and induce tissue plasticity and functional recovery (Nicola et al., 2019). While studies shown that compared with undifferentiated SHED, implantation of induced neural differentiated SHED had a stronger ability to promote nerve regeneration, axonal remyelination, and to inhibit astrocyte generation, glial scar formation in animal models of SCI (Fujii et al., 2015; Taghipour et al., 2012). In our study, SHED sheet was induced with homogenate proteins of spinal cord. The differentiated SHED can continuously secrete cytokines and neurotrophins to promote functional recovery.

Persistent inflammatory cell infiltration after SCI leads to further neural cell death and destruction of cellular and extracellular structures, resulting in the formation of cystic cavities at the lesion. In addition, astrocyte proliferation and ECM deposition promote glial scarring. The formation of cystic cavities and glial scarring after SCI severely hinders nerve regeneration and axonal extension (Alizadeh et al., 2019). During the early stages of SCI, axonal demyelination occurs. The absence of myelin sheath inhibits nerve impulse conduction and affects nerve function (Plemel et al., 2014). Microglia are the resident immune cells of CNS. After SCI, microglia are activated and transformed into macrophages, which colonize the damaged area to phagocytose cell debris and degenerated myelin sheaths (Lin et al., 2021). Microglia phagocytosis of myelin is a feature of neurological disease and injury (Neumann et al., 2009). SCI induces scar formation consisting of multiple cell types (astrocytes, fibroblasts, microglia, macrophages) without spontaneous neuronal regeneration and axonal growth. Hp-SHED sheet group could better promote nerve regeneration and could reduce the inflammatory response and scar formation caused by injury by inhibiting microglia proliferation. Hp-SHED sheet improved the microenvironment by secreting cytokines and neurotrophins to promote nerve regeneration, axonal extension, myelin regeneration and to inhibit astrocyte generation, glial scar formation. Previous studies have shown that hpDCs promote functional recovery from SCI in mice by upregulating neurotrophic cytokines and neurotrophins at the injury site. hpDCs decrease the areas of cysts and exhibit good tissue preservation in SCI mice, which is consistent with our present findings (Liu et al., 2009; Wang et al., 2013).

After spinal cord injury, primary and secondary injuries result in massive neuronal cell death at the lesion as well as destruction of extracellular structures, and a cystic cavity is formed at the injury site. The surviving neural stem cells are more likely to differentiate into astrocytes in the lesion microenvironment, and the proliferation of astrocytes promotes the formation of glial scar (Cao et al., 2001; Wang et al., 2008). The cystic cavity and glial scar formed after SCI severely impede neural regeneration and axonal extension (Alizadeh et al., 2019). Common filler materials can reduce the cystic cavity area by adding exogenous cells or reducing cell death. But the surviving neural stem cells eventually differentiated into astrocytes due to insufficient neurotrophic factors in the lesion microenvironment and formed a glial scar in other studies (Dominguez-Bajo et al., 2019, 2020). Studies have shown that Implantation of SHED with induced neural differentiation has a stronger ability to promote neural regeneration, axonal remyelination and inhibit astrocyte generation and glial scar formation in animal models of SCI (Taghipour et al., 2012; Fujii et al., 2015). Spinal homogenins act as an effective inducer to induce differentiation of stem cells into neuronal cells (Wu et al., 2009; Liu et al., 2011). In this paper, hp-SHED sheet has stronger neurotrophic properties compared to other filler materials. Hp-SHED sheet can inhibit the differentiation of neural stem cells into astrocytes and promote their differentiation into neurons, as evidenced by increased NF expression and decreased GFAP expression at the lesion site. In this way, hp-SHED sheet facilitates the construction of neural networks by reducing glial scar generation and promoting neural axon extension. This is supported by results in other study (Yan et al., 2022).

Nerve regeneration and axonal remyelination promoted the repair and reconstruction of neural networks, which in turn promoted the recovery of sensory and motor functions in SCI rats. Hp-SHED sheet rats had earlier hindlimb sensory repair and earlier recovery of urinary reflex than SHED rats. In addition, the rats in hp-SHED sheet group had better recovery of hindlimb grip strength and better recovery of hindlimb joint functions. Moreover, hp-SHED sheet maintained a favorable safety profile.

Hp-SHED sheet can continuously secrete cytokines and neurotrophins. The neuroprotective factors can improve the pathological microenvironment of spinal cord lesions, promote *in situ* centralis neuroplasticity, allowing regeneration after SCI. Thus, hp-SHED sheet is conducive to enhancing the reconstruction of spinal cord and an ideal bioactive material for filling injuries in the spinal cord.

## 4 Materials and methods

### 4.1 Cell extraction and culture

SHED were obtained from exfoliated deciduous teeth of clinical patients, and teeth were repeatedly flushed with phosphate-buffered saline (PBS). The pulp was removed under aseptic conditions, mechanically sheared, and then digested with type I collagenase (3 mg/mL; Sigma-Aldrich, St. Louis, MO, United States) for 1 h at 37°C to prepare a single-cell suspension. After centrifugation, cells were resuspended in α-Minimum Essential Media (Gibco, Grand Island, NY, United States) containing 10% fetal bovine serum (Hyclone, Logan, UT, United States) and 1% penicillin/streptomycin (Invitrogen, Carlsbad, CA, United States). Cells were incubated in T75 cm^2^ flasks, allowed to grow to 80%–90% confluence, and then passaged normally.

### 4.2 Flow cytometry

Cells in logarithmic growth phase were removed and digested with trypsin into single-cell suspension. Adjust the cell concentration to 1×10^7^ cells/mL. Prepare 6 EP tubes and add 0.1 mL of cell suspensions to each tube. Under light-proof conditions, 1 μl of antibodies were added to each EP tube, Anti-Human CD73 PE, Anti-Human CD105 (Endoglin) PE, Anti-Human/Mouse CD44 APC, Anti-Human CD90 (Thy-1) PE, Anti-Human HLA-DR FITC (BioGems, United States), control group plus PBS. Mix well and incubate for 30 min at 4°C away from light. Vortex shaking was removed every 10 min for 5 s to bring the cells into full contact with the antibody. At the end of incubation, the cells were washed 2–3 times with PBS. Then 0.5 mL of PBS was added to resuspend the cells, filtered through a copper mesh and assayed by flow cytometry (Agilent, United States).

### 4.3 Colony formation assay

SHED in single-cell suspension were seeded into six-well plates at a density of 200 cells per well. Then the cells were cultured in α-MEM at 37°C. The medium was changed every 3 days. The cells were cultured for 10 days. Then the colonies were washed by PBS and fixed in 4% paraformaldehyde (PFA) solution 10 min. After washed by PBS the cells were incubated in 0.1% crystal violet for 20 min (Beyotime, China). Then colonies were observed. Finally, calculate the number of colonies under an inverted light microscope (Leica, Germany).

### 4.4 Osteogenic differentiation

SHED were seeded into 0.1% gelatin-coated six-well plates at a density of 2×10^4^ cells/cm^2^. The cells were cultured in α-MEM at 37°C. When the degree of cell fusion reached 70%, the cells were cultured with osteogenic induction medium (OriCell^®^, China). Cells in control group were cultured in α-MEM. The medium was changed every 3 days. After 21 days of induction, alizarin red was used for staining. The osteogenesis staining was observed under the inverted light microscope.

### 4.5 Adipogenic differentiation

SHED were seeded into 0.1% gelatin-coated six-well plates at a density of 2×10^4^ cells/cm^2^. The cells were cultured in α-MEM at 37°C. When the degree of cell fusion reached 100%, the cells were cultured with adipogenic induction medium A (OriCell^®^, China). The medium was changed to adipogenic induction medium B after 3 days. After 1 day of maintenance, solution B was replaced with solution A. Solution A and solution B are used alternately as above. Cells in control group were cultured in α-MEM. After 28 days of induction, Oil Red O solution was used for staining. The lipid-forming staining was observed under the inverted light microscope.

### 4.6 CCK-8 assay

Cell proliferation was detected with the CCK-8 kit (DOjinDO, Japan). Each 96-well plate was divided into seven groups, with six replicate wells in each group. In six groups, each well was inoculated with 1000 cells in a volume of 200 μL of culture medium with ascorbic acid (AA) concentrations of 0, 15, 30, 60, 120, and 240 μg/mL. The other group was not inoculated with cells, and only the culture medium was added as a blank control. Cells were incubated in a CO_2_ incubator at 37°C. A 96-well plate was removed at 0 h, 24 h, 48 h, 72 h, 96 h and 120 h, and CCK-8 solution (20 μL/well) was added to the culture medium. After incubation for 2 h in the incubator at 37°C, the absorbance was measured at 450 nm.

### 4.7 Preparation of homogenate proteins of spinal cord

Homogenate proteins of the spinal cord were harvested from Sprague-Dawley rats (aged 8–10 weeks) that had been anesthetized. Briefly, spinal cords at the T8–10 levels were resected, ground, filtered (200 mm), disrupted with an ultrasonic homogenizer, and finally centrifuged at 15,000 g for 20 min. The supernatant was collected as the protein homogenate and the protein concentration was measured. The concentration of total protein used here was 1 mg/mL.

### 4.8 Construction of hp-SHED sheet

SHED were cultured in medium containing ascorbic acid (AA), which was changed every 2–3 days. A cell sheet was formed after 1 week of culture. Then, the culture medium was changed to α-MEM containing homogenate proteins of spinal cord for 1 week. Finally, the cell sheet at the bottom of the dish was gently peeled off and curled by self-assembly into a bioactive filler shaped like the spinal cord. This filling material, termed hp-SHED sheet.

### 4.9 Spinal cord injury model and postoperative care

The animal study was reviewed and approved by the Animal Studies Committee of Guangxi University. Twenty female Sprague-Dawley rats, aged 8–10 weeks and weighing 180–230 g, were purchased from the Laboratory Animal Center. In this exploratory study, we utilized female rats, which are commonly used in spinal cord injury studies because of their better compliance with surgical procedures and low rate of self-mutilation compared to males. Besides female rats are superior to male rats in terms of postoperative care due to low urinary infection rate. Rats were housed in animal rooms with sterilized bedding, water, and feed, in a quiet environment with appropriate temperature and humidity. Before the experiment, all rats were subjected to routine behavioral tests to ensure that their motor functions were normal. Rats were numbered sequentially and randomly divided into four groups (*n* = 5 per group): 1) Sham, laminectomy only without spinal cord transection; 2) Ctrl, spinal cord transection with implantation replaced by PBS; 3) SHED, spinal cord transection with SHED implanted at the lesion gap; and 4) hp-SHED sheet, spinal cord transection with hp-SHED sheet implanted at the lesion gap. Rats were fasted for 12 h before surgery and drank water freely. Rats were weighed and anesthetized with 1% pentobarbital sodium (0.4 mL/100 g) by intraperitoneal injection. Under deep anesthesia, each rat was fixed in a prone position on the operating table, and the hair near the T8–T10 spinous process on the back of the rat was shaved, prepared, and positioned to make a posterior median incision centered on T10. At this position, the skin, fascia, and muscle tissues were separated layer by layer to expose the T10 spinous process and transverse process. The T10 spinous process was clipped, the transverse process was held in place with forceps, and the T10 lamina was bitten off with a miniature biting forceps to expose the spinal cord. Care was taken not to touch the spinal cord throughout this process. The spinal cord of the T10 segment was completely transected using ophthalmic scissors. After cutting, the severed end was trimmed slightly to regularize the cut surface for better anastomosis with the filler. The section trimming and the retraction of both rostral and caudal tissue endings produced a gap of 2.0 ± 0.5 mm. Immediately after transection, both lower limbs of the rat were observed to twitch and the rat wagged its tail and then completely relaxed. A filler was implanted into the spinal cord gap to ensure anastomosis with the severed end of the spinal cord. The muscle, fascia, and skin tissues were sutured layer by layer at the end of the operation. The principle of asepsis was strictly observed during the operation. To avoid infection, penicillin was injected intramuscularly daily for 7 days after surgery, and the duration of use was extended in the presence of hematuria and pyuria. Manual urination was performed 2–3 times a day until the urination reflex was restored.

### 4.10 Sensory recovery analysis

Sensory recovery was assessed using the Von Frey test (North Coast Medical, Carlsbad, CA, United States). Briefly, filaments with stimulus force gradients (0.07, 0.4, 2, 4, 10, 60, 180, and 300 g) were applied to the paw to elicit a nociceptive response (rapid stimulus avoidance by the paw) for at least 30 s between trials. Scores were determined by a blinded method. Assessments were performed at 9:00 p.m. due to wide variations in the diurnal activity of rats. Mechanical pain thresholds were assessed using the “Up and Down” method to calculate the 50% retraction-response threshold of rats. Rats with a 50% retraction threshold of 4 g or less were counted as positive for sensory function, and the percentage of positive rats was calculated. The baseline score was measured every 2–3 days after surgery, and then every 20 days until the end of the experiment (60 days).

### 4.11 Grip strength test

Starting 1 week prior to SCI surgery, each rat was trained for 2 min per day to familiarize itself with the grip strength meter (KEWBASIS^®^, KW-ZL-1, China). After 60 days of SCI, the grip strength of the hindlimb was measured for each rat by a blinded method. If the hindlimb motor impairment was too severe for the rat to grasp the crossbar, the grip strength score was 0. The grip strength tests on the same rat were performed a few minutes apart. We made sure that each rat was performed grip test with relaxed muscles and without spasms. Each rat was tested more than three times and the average was taken. The above ensures that the test values are at normal grip levels.

### 4.12 Behavioral evaluation

The Basso-Beattie-Bresnahan (BBB) locomotion rating scale is a neurological assessment method used to evaluate motor function in the hindlimbs of rats. A score of 0 is considered complete paralysis, whereas 21 is considered normal. All animals were checked for bladder fullness prior to the assessment to prevent bladder fullness from interfering with movements. The baseline score was measured every 2–3 days after surgery. Thereafter, measurements were taken every 20 days until the end of the experiment (60 days). In addition, video was recorded for each rat. The results were analyzed and processed using GraphPad Prism 8.0 software (GraphPad, San Diego, CA, United States).

### 4.13 Specimen collection

Sixty days after surgery, rats were anesthetized and systemically perfused with saline. After the saline flow was free of blood, the tissues and organs were fixed with 4% PFA. Subsequently, samples of spinal cord from approximately 2 cm around the lesion site were obtained (including the lesion, as well as the rostral and caudal stumps). Similarly, the liver, spleen, lung, kidney, and bladder were removed and placed in 4% PFA for fixation.

### 4.14 Histological analysis

After self-assembly of hp-SHED sheet, frozen sections were stained with hematoxylin and eosin (HE). Fixed spinal cord samples were cryosectioned, immunofluorescently stained, and imaged by confocal laser-scanning microscope (CLSM) (Leica, Wetzlar, Germany). The bladder was weighed and its volume measured. Finally, the rat liver, spleen, lung, kidney, and bladder were subjected to HE staining. Briefly, PFA-fixed tissues were removed, washed, embedded in paraffin, sectioned, and stained with HE. Images were acquired using an inverted light microscope. Finally, histological analysis was performed.

### 4.15 Immunofluorescence staining

Hp-SHED sheet staining: Hp-SHED sheet was embedded in Optimal Cutting Temperature Compound and cryosectioned at a thickness of 10 μm. Sections were fixed in acetone for 5 min and then washed three times with PBS before incubation at 4°C overnight with one of the following primary antibodies: collagen Ⅰ (Abcam, Cambridge, UK), collagen Ⅲ (Abcam). The next day, an appropriate secondary antibody was added and incubated at room temperature for 1 h, and DAPI was used to stain cell nuclei. Samples were imaged under a CLSM.

Spinal cord staining: PFA-fixed spinal cords were embedded in Optimal Cutting Temperature Compound and sectioned on ice at a thickness of 10 μm. Subsequently, sections were fixed with 4% PFA, washed three times with PBS, and subjected to antigen repair in a cassette filled with EDTA antigen repair buffer (pH 8.0) in a microwave oven. Sections could cool naturally, blocked with 3% bovine serum albumin for 30 min, and incubated overnight at 4°C with one of the following primary antibodies: neurofilament (NF; Cell Signaling Technology), glial fibrillary acidic protein (GFAP; Millipore), calcitonin gene-related peptide (CGRP; GeneTex, Irvine, CA, United States), and myelin basic protein (MBP; Signalway Antibody, Baltimore, MD, United States). The next day, sections were incubated with a secondary antibody at room temperature for 1 h and DAPI was used to restain cell nuclei. After mounting, sections were observed and photographed with a CLSM. Quantitative histomorphometric analysis was performed using Image-Pro Plus software (Media Cybernetics, Rockville, MD, United States).

### 4.16 Statistical analysis

Experimental data were statistically analyzed using GraphPad Prism and SPSS 23.0 software (IBM, Chicago, IL, United States). Measurement data are described as mean ± standard deviation (SD) for n ≥ 3. Differences between two groups were compared utilizing t tests, while those among multiple groups were compared by ANOVA, followed by Tukey’s test for multiple comparisons. BBB scores at different time points were analyzed by repeated measures ANOVA, followed by Tukey’s test for multiple comparisons. *p < 0.05 was considered statistically significant. Significance levels: *p < 0.05, **p < 0.01, ***p < 0.001.

## Data Availability

The original contributions presented in the study are included in the article/Supplementary Material, further inquiries can be directed to the corresponding author.

## Supplementary material

Supplementary Video S1 Locomotion of rats in each group on the 60th day postoperatively.

Supplementary Figure S1 The surgery process of SCI modeling.

Supplementary Figure S2 IF staining of NeuN and Iba1 in Ctrl, SHED and hp SHED sheet.

## References

[B1] Alizadeh A. Dyck S. M. Karimi-Abdolrezaee S. (2019). Traumatic spinal cord injury: An overview of pathophysiology, models and acute injury mechanisms. Front. Neurol. 10, 282. 10.3389/fneur.2019.00282 30967837 PMC6439316

[B2] Anderson M. A. Burda J. E. Ren Y. Ao Y. O'Shea T. M. Kawaguchi R. (2016). Astrocyte scar formation aids central nervous system axon regeneration. Nature 532 (7598), 195–200. 10.1038/nature17623 27027288 PMC5243141

[B3] Cao Q. L. Zhang Y. P. Howard R. M. Walters W. M. Tsoulfas P. Whittemore S. R. (2001). Pluripotent stem cells engrafted into the normal or lesioned adult rat spinal cord are restricted to a glial lineage. Exp. Neurol. 167 (1), 48–58. 10.1006/exnr.2000.7536 11161592

[B4] Chen Y. R. Lai P. L. Chien Y. Lee P. H. Lai Y. H. Ma H. I. (2020). Improvement of impaired motor functions by human dental exfoliated deciduous teeth stem cell-derived factors in a rat model of Parkinson's disease. Int. J. Mol. Sci. 21 (11), 3807. 10.3390/ijms21113807 32471263 PMC7312764

[B5] Dias D. O. Kim H. Holl D. Werne S. B. Lundeberg J. Carlen M. (2018). Reducing pericyte-derived scarring promotes recovery after spinal cord injury. Cell 173 (1), 153–165.e22. 10.1016/j.cell.2018.02.004 29502968 PMC5871719

[B6] Dominguez-Bajo A. Gonzalez-Mayorga A. Guerrero C. R. Palomares F. J. Garcia R. Lopez-Dolado E. (2019). Myelinated axons and functional blood vessels populate mechanically compliant rgo foams in chronic cervical hemisected rats. Biomaterials 192, 461–474. 10.1016/j.biomaterials.2018.11.024 30502723

[B7] Dominguez-Bajo A. Gonzalez-Mayorga A. Lopez-Dolado E. Munuera C. Garcia-Hernandez M. Serrano M. C. (2020). Graphene oxide microfibers promote regenerative responses after chronic implantation in the cervical injured spinal cord. ACS Biomater. Sci. Eng. 6 (4), 2401–2414. 10.1021/acsbiomaterials.0c00345 33455347

[B8] Elloumi-Hannachi I. Yamato M. Okano T. (2010). Cell sheet engineering: A unique nanotechnology for scaffold-free tissue reconstruction with clinical applications in regenerative medicine. J. Intern. Med. 267 (1), 54–70. 10.1111/j.1365-2796.2009.02185.x 20059644

[B9] Fan Z. Liao X. Tian Y. Xuzhuzi X. Nie Y. (2020). A prevascularized nerve conduit based on a stem cell sheet effectively promotes the repair of transected spinal cord injury. Acta Biomater. 101, 304–313. 10.1016/j.actbio.2019.10.042 31678739

[B10] Fujii H. Matsubara K. Sakai K. Ito M. Ohno K. Ueda M. (2015). Dopaminergic differentiation of stem cells from human deciduous teeth and their therapeutic benefits for parkinsonian rats. Brain Res. 1613, 59–72. 10.1016/j.brainres.2015.04.001 25863132

[B11] Global R. Abate D. Abate K. H. Abay S. M. Abbafati C. Abbasi N. (2018). Global, regional, and national incidence, prevalence, and years lived with disability for 354 diseases and injuries for 195 countries and territories, 1990–2017: A systematic analysis for the global burden of disease study 2017. Lancet 392 (10159), 1789–1858. 10.1016/S0140-6736(18)32279-7 30496104 PMC6227754

[B12] Hochuli A. Senegaglia A. C. Selenko A. H. Fracaro L. Brofman P. (2021). Dental pulp from human exfoliated deciduous teeth-derived stromal cells demonstrated neuronal potential: *In vivo* and *in vitro* studies. Curr. Stem Cell Res. Ther. 16 (5), 495–506. 10.2174/1574888X16666210215160402 33588741

[B13] Huang G. T. Gronthos S. Shi S. (2009). Mesenchymal stem cells derived from dental tissues vs. Those from other sources: Their biology and role in regenerative medicine. J. Dent. Res. 88 (9), 792–806. 10.1177/0022034509340867 19767575 PMC2830488

[B14] Huang L. Zheng Z. Bai D. Han X. (2022). Stem cells from human exfoliated deciduous teeth and their promise as preventive and therapeutic strategies for neurological diseases and injuries. Curr. Stem Cell Res. Ther. 17 (6), 527–536. 10.2174/1574888X17666211229155533 34967291

[B15] Hwang N. S. Zhang C. Hwang Y. S. Varghese S. (2009). Mesenchymal stem cell differentiation and roles in regenerative medicine. Wiley Interdiscip. Rev.-Syst. Biol. 1 (1), 97–106. 10.1002/wsbm.26 20835984

[B16] Iwata T. Yamato M. Washio K. Yoshida T. Tsumanuma Y. Yamada A. (2018). Periodontal regeneration with autologous periodontal ligament-derived cell sheets - a safety and efficacy study in ten patients. Regen. Ther. 9, 38–44. 10.1016/j.reth.2018.07.002 30525074 PMC6222282

[B17] Li L. Xiao B. Mu J. Zhang Y. Zhang C. Cao H. (2019). A mno2 nanoparticle-dotted hydrogel promotes spinal cord repair via regulating reactive oxygen species microenvironment and synergizing with mesenchymal stem cells. ACS Nano 13 (12), 14283–14293. 10.1021/acsnano.9b07598 31769966

[B18] Li X. Dai J. (2018). Bridging the gap with functional collagen scaffolds: Tuning endogenous neural stem cells for severe spinal cord injury repair. Biomater. Sci. 6 (2), 265–271. 10.1039/c7bm00974g 29265131

[B19] Lin S. Zhou Z. Zhao H. Xu C. Guo Y. Gao S. (2021). Tnf promotes m1 polarization through mitochondrial metabolism in injured spinal cord. Free Radic. Biol. Med. 172, 622–632. 10.1016/j.freeradbiomed.2021.07.014 34252538

[B20] Lin Y. C. Grahovac T. Oh S. J. Ieraci M. Rubin J. P. Marra K. G. (2013). Evaluation of a multi-layer adipose-derived stem cell sheet in a full-thickness wound healing model. Acta Biomater. 9 (2), 5243–5250. 10.1016/j.actbio.2012.09.028 23022891

[B21] Liu M. Zhao J. Liang H. Bian X. (2009). Vaccination with dendritic cells pulsed with homogenate protein of spinal cord promotes functional recovery from spinal cord injury in mice. Spinal Cord. 47 (5), 360–366. 10.1038/sc.2008.112 18825159

[B22] Liu R. Fan D. Jin P. Fan H. Wang P. (2011). Effects of injured spinal cord extracts on brain-derived neurotropic factor and myelin proteolipid protein in bone marrow mesenchymal stem cells. Chin. J. Tissue Eng. Res. 15 (1), 7.

[B23] Liu Y. Ming L. Luo H. Liu W. Zhang Y. Liu H. (2013). Integration of a calcined bovine bone and bmsc-sheet 3d scaffold and the promotion of bone regeneration in large defects. Biomaterials 34 (38), 9998–10006. 10.1016/j.biomaterials.2013.09.040 24079891

[B24] Miura M. Gronthos S. Zhao M. Lu B. Fisher L. W. Robey P. G. (2003). Shed: Stem cells from human exfoliated deciduous teeth. Proc. Natl. Acad. Sci. U. S. A. 100 (10), 5807–5812. 10.1073/pnas.0937635100 12716973 PMC156282

[B25] Moalem G. Gdalyahu A. Shani Y. Otten U. Lazarovici P. Cohen I. R. (2000). Production of neurotrophins by activated t cells: Implications for neuroprotective autoimmunity. J. Autoimmun. 15 (3), 331–345. 10.1006/jaut.2000.0441 11040074

[B26] Neumann H. Kotter M. R. Franklin R. J. (2009). Debris clearance by microglia: An essential link between degeneration and regeneration. Brain 132, 288–295. 10.1093/brain/awn109 18567623 PMC2640215

[B27] Nicola F. Marques M. R. Odorcyk F. Arcego D. M. Petenuzzo L. Aristimunha D. (2017). Neuroprotector effect of stem cells from human exfoliated deciduous teeth transplanted after traumatic spinal cord injury involves inhibition of early neuronal apoptosis. Brain Res. 1663, 95–105. 10.1016/j.brainres.2017.03.015 28322752

[B28] Nicola F. Marques M. R. Odorcyk F. Petenuzzo L. Aristimunha D. Vizuete A. (2019). Stem cells from human exfoliated deciduous teeth modulate early astrocyte response after spinal cord contusion. Mol. Neurobiol. 56 (1), 748–760. 10.1007/s12035-018-1127-4 29796991

[B29] Nishida K. Yamato M. Hayashida Y. Watanabe K. Yamamoto K. Adachi E. (2004). Corneal reconstruction with tissue-engineered cell sheets composed of autologous oral mucosal epithelium. N. Engl. J. Med. 351 (12), 1187–1196. 10.1056/NEJMoa040455 15371576

[B30] Ohki T. Yamato M. Ota M. Takagi R. Murakami D. Kondo M. (2012). Prevention of esophageal stricture after endoscopic submucosal dissection using tissue-engineered cell sheets. Gastroenterology 143 (3), 582–588.e2. 10.1053/j.gastro.2012.04.050 22561054

[B31] Pereira L. V. Bento R. F. Cruz D. B. Marchi C. Salomone R. Oiticicca J. (2019). Stem cells from human exfoliated deciduous teeth (shed) differentiate *in vivo* and promote facial nerve regeneration. Cell Transpl. 28 (1), 55–64. 10.1177/0963689718809090 PMC6322138 30380914

[B32] Plemel J. R. Keough M. B. Duncan G. J. Sparling J. S. Yong V. W. Stys P. K. (2014). Remyelination after spinal cord injury: Is it a target for repair? Prog. Neurobiol. 117, 54–72. 10.1016/j.pneurobio.2014.02.006 24582777

[B33] Rubiano A. M. Carney N. Chesnut R. Puyana J. C. (2015). Global neurotrauma research challenges and opportunities. Nature 527 (7578), S193–S197. 10.1038/nature16035 26580327

[B34] Sakai K. Yamamoto A. Matsubara K. Nakamura S. Naruse M. Yamagata M. (2012). Human dental pulp-derived stem cells promote locomotor recovery after complete transection of the rat spinal cord by multiple neuro-regenerative mechanisms. J. Clin. Invest. 122 (1), 80–90. 10.1172/JCI59251 22133879 PMC3248299

[B35] Sato M. Yamato M. Hamahashi K. Okano T. Mochida J. (2014). Articular cartilage regeneration using cell sheet technology. Anat. Rec. 297 (1), 36–43. 10.1002/ar.22829 24293096

[B36] Shimizu T. Yamato M. Kikuchi A. Okano T. (2003). Cell sheet engineering for myocardial tissue reconstruction. Biomaterials 24 (13), 2309–2316. 10.1016/s0142-9612(03)00110-8 12699668

[B37] Sugimura-Wakayama Y. Katagiri W. Osugi M. Kawai T. Ogata K. Sakaguchi K. (2015). Peripheral nerve regeneration by secretomes of stem cells from human exfoliated deciduous teeth. Stem Cells Dev. 24 (22), 2687–2699. 10.1089/scd.2015.0104 26154068 PMC4652186

[B38] Taghipour Z. Karbalaie K. Kiani A. Niapour A. Bahramian H. Nasr-Esfahani M. H. (2012). Transplantation of undifferentiated and induced human exfoliated deciduous teeth-derived stem cells promote functional recovery of rat spinal cord contusion injury model. Stem Cells Dev. 21 (10), 1794–1802. 10.1089/scd.2011.0408 21970342

[B39] Takeuchi R. Kuruma Y. Sekine H. Dobashi I. Yamato M. Umezu M. (2016). *In vivo* vascularization of cell sheets provided better long-term tissue survival than injection of cell suspension. J. Tissue Eng. Regen. Med. 10 (8), 700–710. 10.1002/term.1854 24470393

[B40] Tang S. W. Tong W. Y. Pang S. W. Voelcker N. H. Lam Y. W. (2020). Deconstructing, replicating, and engineering tissue microenvironment for stem cell differentiation. Tissue Eng. Part b-rev. 26 (6), 540–554. 10.1089/ten.TEB.2020.0044 32242476

[B41] Thuret S. Moon L. D. Gage F. H. (2006). Therapeutic interventions after spinal cord injury. Nat. Rev. Neurosci. 7 (8), 628–643. 10.1038/nrn1955 16858391

[B42] Tran A. P. Warren P. M. Silver J. (2018). The biology of regeneration failure and success after spinal cord injury. Physiol. Rev. 98 (2), 881–917. 10.1152/physrev.00017.2017 29513146 PMC5966716

[B43] Wang B. Xiao Z. Chen B. Han J. Gao Y. Zhang J. (2008). Nogo-66 promotes the differentiation of neural progenitors into astroglial lineage cells through mtor-stat3 pathway. PLoS One 3 (3), e1856. 10.1371/journal.pone.0001856 18365011 PMC2266802

[B44] Wang K. Chao R. Guo Q. N. Liu M. Y. Liang H. P. Liu P. (2013). Expressions of some neurotrophins and neurotrophic cytokines at site of spinal cord injury in mice after vaccination with dendritic cells pulsed with homogenate proteins. Neuroimmunomodulation 20 (2), 87–98. 10.1159/000345522 23257628

[B45] Wang L. Gu S. Gan J. Tian Y. Zhang F. Zhao H. (2021). Neural stem cells overexpressing nerve growth factor improve functional recovery in rats following spinal cord injury via modulating microenvironment and enhancing endogenous neurogenesis. Front. Cell. Neurosci. 15, 773375. 10.3389/fncel.2021.773375 34924958 PMC8675903

[B46] Wu J. Li L. X. Wu X. (2009). An experimental study on neuron-like cells from mesenchymal stem cells induced by spinal cord supernatant. Prog. Mod. Biomed. 9 (06), 1085–1088.

[B47] Yamazaki K. Kawabori M. Seki T. Takamiya S. Konno K. Watanabe M. (2021). Mesenchymal stem cell sheet promotes functional recovery and palliates neuropathic pain in a subacute spinal cord injury model. Stem Cells Int. 2021, 1–18. 10.1155/2021/9964877 PMC8285204 34306098

[B48] Yan J. Zhang L. Li L. He W. Liu W. (2022). Developmentally engineered bio-assemblies releasing neurotrophic exosomes guide *in situ* neuroplasticity following spinal cord injury. Mat. Today Bio 16, 100406. 10.1016/j.mtbio.2022.100406 PMC9440432 36065352

[B49] Yang J. Yamato M. Kohno C. Nishimoto A. Sekine H. Fukai F. (2005). Cell sheet engineering: Recreating tissues without biodegradable scaffolds. Biomaterials 26 (33), 6415–6422. 10.1016/j.biomaterials.2005.04.061 16011847

[B50] Yu W. R. Fehlings M. G. (2011). Fas/fasl-mediated apoptosis and inflammation are key features of acute human spinal cord injury: Implications for translational, clinical application. Acta Neuropathol. 122 (6), 747–761. 10.1007/s00401-011-0882-3 22038545 PMC3224722

