# Supplementary figures and images for: Implantation with SHED sheet induced with homogenate protein of spinal cord promotes functional recovery from spinal cord injury in rats
Source: Front Bioeng Biotechnol. 2023 Mar 14;11:1119639. doi: 10.3389/fbioe.2023.1119639 (PMC10043224; doi:10.3389/fbioe.2023.1119639)

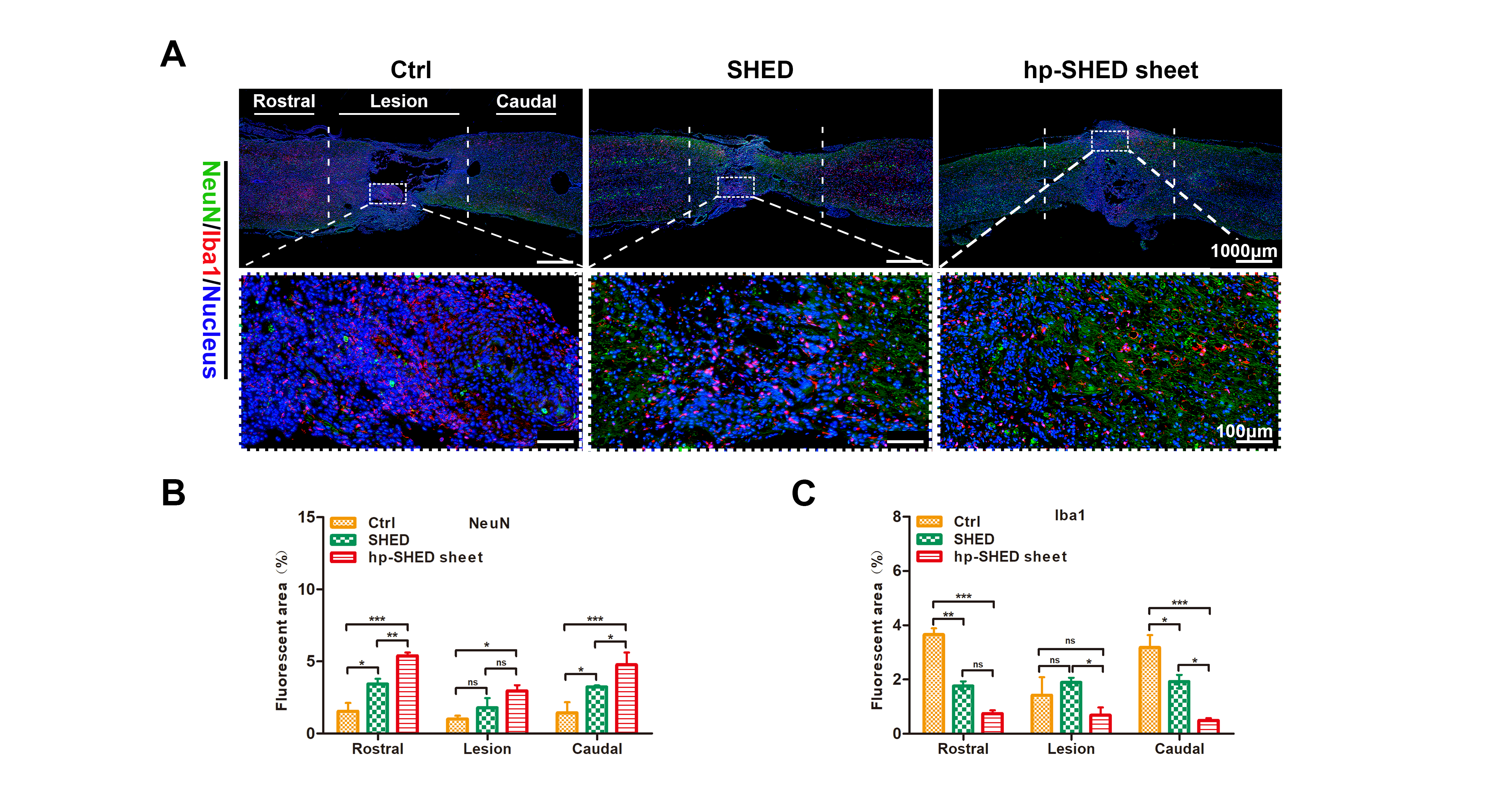

Supplement: Supplementary file 1 [file Image2.TIF]

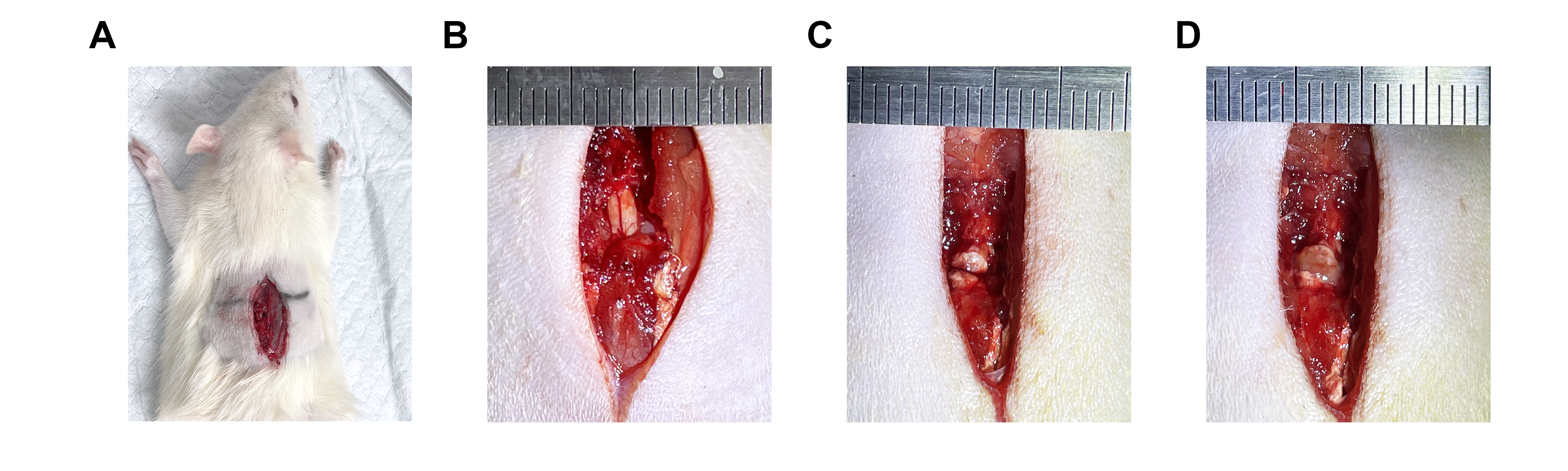

Supplement: Supplementary file 2 [file Image1.TIF]
